# Complete Genome Sequence for *Lactobacillus helveticus* CNRZ 32, an Industrial Cheese Starter and Cheese Flavor Adjunct

**DOI:** 10.1128/genomeA.00590-13

**Published:** 2013-08-22

**Authors:** Jeff R. Broadbent, Joanne E. Hughes, Dennis L. Welker, Thomas A. Tompkins, James L. Steele

**Affiliations:** Department of Nutrition, Dietetics, and Food Sciences, Utah State University, Logan, Utah, USAa; Department of Biology, Utah State University, Logan, Utah, USAb; Institut Rosell, Montreal, QC, Canadac; Department of Food Science, University of Wisconsin-Madison, Madison, Wisconsin, USAd

## Abstract

*Lactobacillus helveticus* is a lactic acid bacterium widely used in the manufacture of cheese and for production of bioactive peptides from milk proteins. We present the complete genome sequence for *L. helveticus* CNRZ 32, a strain particularly recognized for its ability to reduce bitterness and accelerate flavor development in cheese.

## GENOME ANNOUNCEMENT

Among lactic acid bacteria, *Lactobacillus helveticus* is recognized for its active protease and peptidase activities toward milk proteins (1–4), and the species is used worldwide as a starter culture in the manufacture of cheeses and for the production of bioactive peptides from milk proteins during fermentation (5, 6). The commercial strain *L. helveticus* CNRZ 32 is widely used to reduce bitterness and accelerate flavor development in cheese and has also been shown to release bioactive peptides in milk (7–9). Because these properties are strain specific (10–15), comparative genomic studies of *L. helveticus* should reveal mechanisms to more efficiently utilize these organisms in food and health.

The *L. helveticus* CNRZ 32 genome sequence was determined by random shotgun sequencing data from three separate small (1- to 2.5-kb) insert libraries produced at the University of Wisconsin Genome Center (Madison, WI), Lucigen, Inc. (Madison, WI), and the U.S. Department of Energy Joint Genome Institute (JGI) (Walnut Creek, CA) as well as data from a large (10- to 20-kb) insert library developed at Lucigen, Inc. Random clones from libraries generated at the Wisconsin Genome Center and Lucigen were sequenced by using dye-terminator chemistry on Applied Biosystems ABI377 and 3700 automated sequencers, while the JGI library was sequenced via dye-terminator chemistry on PerkinElmer (Foster City, CA) PE 377 automated DNA sequencers. Sequence data from all three libraries were assembled with DNASTAR (Madison, WI) SeqMan II software (version 5.08). Gap closure and sequence polishing were performed via PCR to ensure at least a 4-fold level of unambiguous, bidirectional nucleotide sequence data. The genome was computer annotated using the ERGO bioinformatics suite (Integrated Genomics, Chicago, IL) and manually curated. Finally, correct assembly of the *L. helveticus* CNRZ 32 genome was verified by alignment with a high-resolution, whole-genome optical NheI restriction map (16) derived from this strain (OpGen, Inc., Gaithersburg, MD).

The *L. helveticus* CNRZ 32 genome consists of a single circular chromosome of 2,225,962 bp that is predicted to carry 1,685 genes, including 63 tRNA genes and 4 rRNA operons. The genome includes 163 predicted pseudogenes (excluding transposases) and 356 complete or partial insertion sequence (IS) elements. The large number of pseudogenes and IS elements is consistent with a previous report for *L. helveticus* DPC 4571 and supports the hypothesis that this species has experienced significant genome decay (17).

Comparison against the complete genomes of *L. helveticus* DPC 4571 (17), *L. helveticus* H10 (18), and *L. helveticus* R0052 (19) indicated that CNRZ 32 contained over 180 predicted coding sequences that were not present in any of the other sequenced *L. helveticus* strains. Examples of particular interest include a unique gene cluster for biosynthesis of a phosphocholine-modified exopolysaccharide and several gene clusters that indicate that CNRZ 32 harbors at least one prophage. Comparative genomics also confirmed that CNRZ 32 is the only sequenced *L. helveticus* strain to carry genes encoding four distinct cell envelope-associated proteinases (20).

### Nucleotide sequence accession number. 

Nucleotide sequence data for the complete *L. helveticus* CNRZ 32 genome with corresponding gene annotations have been deposited in GenBank under accession number CP002081. The version described in this paper is the first version.
